# Organic acids and microencapsulated phenolic compounds in diets for laying hens

**DOI:** 10.1016/j.psj.2026.106895

**Published:** 2026-04-02

**Authors:** Paloma Eduarda Lopes de Souza, Adiel Vieira de Lima, Carlos Henrique do Nascimento, Aline Beatriz Rodrigues, Amanda Fabrício Dantas de Lima, Cleber Franklin Santos de Oliveira, Edijanio Galdino da Silva, Apolônio Gomes Ribeiro, Ricardo Romão Guerra, Danilo Teixeira Cavalcante, Isabelle Naemi Kaneko, Andreia Massuquetto, Thiago Pereira Ribeiro, Lucas Rannier Ribeiro Antonino Carvalho, Fernando Guilherme Perazzo Costa

**Affiliations:** a Department of Animal Science, Federal University of Paraiba, Highway PB-079, Areia, PB, 58397-000, Brazil; b Department of Statistics, Federal University of Alfenas (UNIFAL-MG), Rua Gabriel Monteiro da Silva, 700, Centro, Alfenas, MG, Brazil; c Department of Veterinary Sciences, Federal University of Paraíba (UFPB), Areia, PB, Brazil; d Department of Animal Science, Federal University of Agreste of Pernambuco, Bom Pastor Avenue, no number, Boa Vista, Garanhuns, PE, 55292-270, Brazil; e Department of Animal Science, Federal University of Rondônia (UNIR), Brazil; f Tectron – Technology and Innovation, Toledo, PR, 85902-010, Brazil; g Department of Physiology and Pharmacology, Karolinska Institutet, Biomedicum 5B, Solnavägen 9, S-171 77, Stockholm, Sweden

**Keywords:** Natural additives, Antioxidants, Encapsulation, Intestinal health

## Abstract

This study evaluated the effects of dietary supplementation with microencapsulated organic acids and phenolic compounds in laying hens. The experiment was conducted with 500 Novogen Brown laying hens from 30 weeks of age. Birds were assigned to five treatments with 10 replicates of 10 birds each in a completely randomized design. The treatments consisted of: CD = control diet; 200AD1 = 200 g/t additive 1; 500AD1 = 500 g/t additive 1; 200AD2 = 200 g/t additive 2; and 500AD2 = 500 g/t additive 2. Productive performance, egg quality, organ pH and relative organ weight, intestinal histomorphometry, and serum biochemical parameters were evaluated. Treatment means were compared using the Tukey test at a 5% significance level. Dietary supplementation affected productive performance, egg quality, gastrointestinal morphology, and selected serum biochemical indicators. Birds receiving 500AD1 and 500AD2 showed higher egg production compared with those receiving 200AD2, whereas egg weight was lower in 500AD2 compared with 200AD1. Egg quality traits were also influenced, with greater eggshell thickness observed in 200AD2 and improved yolk index in 200AD2 and 500AD2 compared with the control diet. Relative weights of digestive organs, including gizzard, small intestine, pancreas, and proventriculus, were generally higher in birds receiving 200AD2. Cecal pH was reduced in 500AD1 compared with the other treatments. Intestinal histomorphometry showed significant alterations in villus structure, absorptive area, and goblet cell counts across intestinal segments, with 500AD2 frequently associated with higher villus:crypt ratios and greater ileal villus height. Serum biochemical parameters were also affected, including uric acid, γ-glutamyl transferase, calcium, and phosphorus. In summary, supplementation with microencapsulated organic acids and phenolic compounds modulated productive performance, egg quality, and intestinal physiology in laying hens. Higher inclusion levels, particularly 500AD2, were associated with more favorable intestinal morphometric responses and productive outcomes, whereas 200AD2 appeared to promote structural adaptations of the gastrointestinal tract. These findings highlight the potential of microencapsulated acidifier phytogenic blends as nutritional strategies to support intestinal functionality and productive efficiency in laying hens.

## Introduction

In recent years, research efforts have intensified to identify innovative alternatives to antibiotics, which are associated with antimicrobial resistance. In this context, natural compounds have emerged as promising options, particularly organic acids and essential oils (Islam et al., 2022). These compounds influence productive performance by increasing nutrient digestibility and absorption, improving intestinal morphology by promoting villus development, and modulating the microbiota by increasing beneficial microorganisms; moreover, their combination can amplify these effects (Stamilla et al., 2020).

Essential oils (EO) contain high concentrations of bioactive phenolic compounds that exert antimicrobial, antioxidant, and anti-inflammatory effects in the ileum through the regulation of gene expression in this tissue (Moharreri et al., 2021). Commonly used essential oils include carvacrol, thymol, eugenol, cinnamaldehyde, palm oil, among other phytogenic compounds. Orzuna-Orzuna and Lara-Bueno (2023) reported that dietary inclusion of essential oils increased serum levels of the antioxidant enzymes superoxide dismutase and glutathione peroxidase, along with total antioxidant capacity. Kolani et al. (2019) reported that adding 2% palm oil resulted in beneficial effects on egg production performance in laying hens.

Organic acids are weak acids that partially dissociate and can act as antibacterial agents, immune enhancers, and growth promoters (Khan et al., 2022). Acidification of the intestine inhibits pathogenic bacteria that compete with the host for available nutrients and reduces the production of toxic bacterial metabolites (Palhares and Kunz, 2011). Short-chain fatty acids, medium-chain fatty acids, and other organic acids exhibit different levels of antimicrobial activity depending on both acid concentration and the bacterial species exposed (Khan and Iqbal, 2016). Organic acids belong to different classes (butyric, acetic, citric, formic, lactic, propionic, among others), and in addition to their antimicrobial action, they stimulate enzymatic activity (Funari Junior et al., 2015).

Due to their volatility, organic acids and essential oils can evaporate during feed processing. Furthermore, their action is often restricted to the stomach and proximal small intestine. These factors can limit their overall biological efficacy (Michiels et al., 2008). Microencapsulation has therefore been developed as a technological strategy to enhance the stability of these compounds during feed processing and to enable their controlled release along the gastrointestinal tract (Jadhao et al., 2019). In this process, active compounds are coated with a lipid-based matrix, allowing them to remain relatively inactive in the stomach and to be released under the action of bile salts and lipases in the intestine, where their biological effects are expected to occur. Abdelli et al. (2021) demonstrated that microencapsulation protected fumaric acid and thymol, allowing their gradual release throughout the gastrointestinal tract.

Despite the growing body of literature on organic acids and essential oils, most studies have evaluated these additives individually or have focused primarily on productive performance outcomes (Cai et al., 2025; Chen et al., 2025; Waghmare et al., 2025). Comparative investigations assessing different microencapsulated formulations and their combined effects on productive performance, intestinal morphology, digestive tract development, and metabolic responses in laying hens remain limited. Moreover, information regarding how distinct microencapsulated formulations may differentially influence physiological and biochemical responses in laying hens is still scarce.

Therefore, the objective of this study was to compare the effects of different microencapsulated organic acid– and essential oil-based additives on productive performance, egg quality, digestive tract pH and development, intestinal morphology, and serum biochemical parameters in laying hens.

## Materials and methods

The experimental protocol was approved by the Animal Ethics Committee (CEUA) of the Federal University of Paraíba, Brazil, under approval certificate No. 7935130325/2025.

### Experimental site, design, and diet

The experiment was conducted at the Poultry Module of the Department of Animal Science, Center of Agricultural Sciences, Federal University of Paraíba (CCA/UFPB), Areia, PB, Brazil. Environmental conditions, including temperature and relative humidity, were recorded daily throughout the experiment, with average values of 23.8°C and 82% RH.

A total of 500 Novogen Brown laying hens from 30 weeks of age were used. Birds were distributed into six treatments, with 10 replicates of 10 birds each, in a completely randomized design. The experimental period lasted 112 days, divided into four 28-day cycles, during which productive performance and egg quality were evaluated in each cycle.

**Fig. 1:**
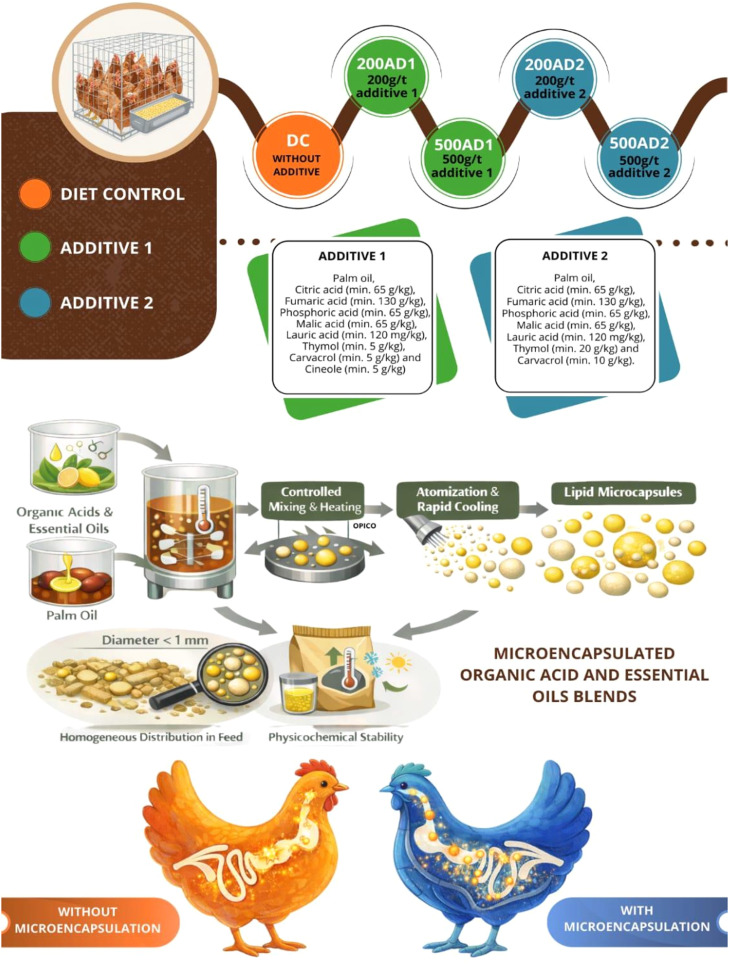
Description of the treatments and additives used. Representation of the treatments: control diet (CD) without additive; 200 and 500 g/t of additive 1 (200AD1 and 500AD1); 200 and 500 g/t of additive 2 (200AD2 and 500AD2). The additives contain mixtures of organic acids and phenolic compounds.

The treatments were as follows: CD = control diet, 200AD1 = 200 g/t additive 1, 500AD1 = 500 g/t additive 1, 200AD2 = 200 g/t additive 2, 500AD2 = 500 g/t additive 2 (Fig. 1).

Diets (Table 1) were formulated according to the requirements described in the strain management guide, and additives were included at the corresponding levels for each treatment (on-top inclusion in a common basal diet, without changes in dietary nutrient levels). Feed was supplied daily at 7:00 a.m. and 4:00 p.m.

**Table 1:** Composition of experimental diets for laying hens during the production period.

Ingredients	kg	Nutritional Levels	
Corn	567.578	Metabolizable energy, kcal/kg	2900
Soybean meal	286.899	Crude protein, %	17.500
Soybean oil	34.102	Crude fat, %	5.916
Fine limestone	42.690	Crude fiber, %	2.454
Coarse limestone	42.690	Calcium, %	4.000
Dicalcium phosphate	17.757	Available phosphorus, %	0.420
Sodium bicarbonate	0.804	Digestible lysine, %	0.812
Salt	3.093	Digestible methionine, %	0.442
L-Lysine (80%)	0.195	Digestible methionine + cysteine, %	0.674
DL-Methionine (98%)	2.037	Digestible threonine, %	0.581
L-Threonine (98%)	0.298	Digestible tryptophan, %	0.192
Bentonite (Adsorbent)	1.000	Choline, mg/kg	1200
Mineral premix^1^	0.500	Sodium, %	0.180
Vitamin premix^2^	0.250	Chlorine, %	0.240
Choline chloride (60%)	0.108	Na+K–Cl, mEq/kg	188
Total	1000		

1 Supplied (per kilogram of diet): 66 mg of Fe (FeSO4·7H2O), 83 mg of Zn (ZnSO4·7H2O), 80 mg of Mn (MnSO4·H2O), 1 mg of I (KI), and 6.8 mg of Cu (CuSO4·5H2O).

2 Supplied (per kilogram of diet): 11,700 IU of vitamin A; 3,600 IU of vitamin D3; 21 IU of vitamin E; 4.2 mg of vitamin K3; 3.0 mg of vitamin B1; 10.2 mg of vitamin B2; 0.9 mg of folic acid; 15 mg of calcium pantothenate; 45 mg of niacin; 5.4 mg of vitamin B6; 24 μg of vitamin B12; and 150 μg of biotin.

The acid-regulating additives included in the diets contained the following compositions:

1. Additive 1 – Palm oil, citric acid (min.) 65 g/kg, fumaric acid (min.) 130 g/kg, phosphoric acid (min.) 65 g/kg, malic acid (min.) 65 g/kg, lauric acid (min.) 120 mg/kg, thymol (min.) 5 g/kg, carvacrol (min.) 5 g/kg, cineole (min.) 5 g/kg.
2. Additive 2 – Palm oil, citric acid (min.) 65 g/kg, fumaric acid (min.) 130 g/kg, phosphoric acid (min.) 65 g/kg, malic acid (min.) 65 g/kg, lauric acid (min.) 120 mg/kg, thymol (min.) 20 g/kg, carvacrol (min.) 10 g/kg.

The evaluated products were microencapsulated organic acid and essential oils blends (Microacid Eoils and Microacid Eoils Plus, Tectron. Brazil). The active compounds were encapsulated within a palm oil–based lipid matrix using a proprietary thermo-mechanical microencapsulation process. Briefly, the organic acids were dispersed in molten palm oil under controlled temperature and agitation, followed by atomization and rapid cooling to form solid lipid microcapsules. The resulting particles presented a mean diameter below 1 mm, ensuring homogeneous distribution in the feed. The lipid coating is designed to enhance physicochemical stability during feed processing and storage, while modulating the release of active compounds along the gastrointestinal tract

### Productive performance

Productive performance was evaluated throughout all experimental cycles based on egg production, egg weight (g), feed intake (g/bird/day), egg mass, feed conversion per dozen eggs (kg/dozen), feed conversion per egg mass (kg/kg), and viability (number of birds present ÷ number of birds housed × 100).

Egg production (%) was calculated as the number of eggs produced divided by the number of birds housed, multiplied by 100, with production monitored daily. Feed intake was determined by the difference between the feed offered at the beginning of each cycle and the weight of feed leftovers at the end of each cycle, adjusted for mortality to reflect actual feed consumption. Feed conversion per dozen eggs was calculated by dividing feed intake by the number of dozens of eggs produced and corrected for bird mortality.

Egg weight was determined by collecting and individually weighing all eggs from each replicate using an analytical scale (Model AUW220, Shimadzu do Brasil, São Paulo, Brazil) with a precision of 0.0001 g (four-decimal scale). Egg mass was calculated by multiplying egg production by the mean egg weight for each replicate over the period. Feed conversion per egg mass (kg feed/kg egg) was calculated as the ratio of feed intake to egg mass produced for each replicate over the period.

### Egg quality

Egg quality evaluations were performed during the last three days of each cycle, considering three eggs per replicate. Parameters assessed included egg weight, yolk centering, specific gravity, yolk, albumen, and shell percentages, shell thickness (mm), yolk color, Haugh unit, yolk pH, and albumen pH.

Specific gravity was determined based on Archimedes’ principle as described by Freitas et al. (2004), and the SG value was obtained using the equation:

$$SG=\frac{egg\;weight}{egg\;weight\;in\;water\;\times \;temperature\;correction}$$

Water density correction based on temperature was calculated as:

D = (0,9998676 + 17,801161 × 10-3t −7,942501 × 10-6t - 52,56328 × 10-9t + 137,6891 × 10-12t - 364,4647 × 10-15t) / (1 + 17,735441 × 10-3t), where t is the temperature in degrees Celsius (Kell, 1975).

Albumen quality was evaluated by determining the Haugh unit. Eggs were broken onto a flat surface, and the height (mm) of the thick albumen was measured using a digital caliper (Model 100.174BL, Manufacturer: Digimess, São Paulo, Brazil). The Haugh unit was calculated using the equation:

$$HU=100\text{log}\left(H-1.7\;\times P^{0.37}+7.6\right)$$

where HU = Haugh unit, H = albumen height in mm, and P = egg weight in g.

Next, the yolk was separated from the albumen and weighed using a semi-analytical balance (0.0001 g) (Model LS10, Manufacturer: Marte Científica, Minas Gerais, Brazil). Yolk percentage was calculated as the yolk weight divided by the egg weight, multiplied by 100. Albumen percentage was obtained by difference:

%albumen=100−(%yolk+%shell)

Albumen weight was calculated as the difference between egg weight and the sum of yolk and shell weights.

Yolk color was determined using the DSM YolkFan™ colorimetric scale (Model dsm-firmenich YolkFan™, Manufacturer: DSM-firmenich, Geneva, Switzerland). The yolk index, defined as the ratio of yolk height to yolk width, was measured with a digital caliper (Model 100.174BL, Manufacturer: Digimess, São Paulo, Brazil). Yolk and albumen pH were determined using a portable digital pH meter (Model AKSO-AK90, Manufacturer: AKSO, Rio Grande do Sul, Brazil). Yolk centering was assessed using a visual scoring system (1–10) (Wesley and Stadelman, 1959).

Eggshells were identified, dried at room temperature for one week, and weighed on a digital (Model 100.174BL, Manufacturer: Digimess, São Paulo, Brazil) scale with 0.0001 g precision to obtain the average shell weight. Shell percentage was calculated as the average shell weight divided by the average egg weight, multiplied by 100. Shell thickness was measured at the equatorial region using a digital micrometer, 0–25 mm, 0.001 mm precision (Model 293-340-32, Manufacturer: Mitutoyo, São Paulo, Brazil), with the reported thickness being the average of three points measured on each egg.

### Organ weights and pH

To evaluate the development of the digestive tract, organ weights (gizzard, liver, small intestine, large intestine, pancreas, proventriculus, and spleen) and abdominal fat were measured at the end of the fourth experimental cycle. Birds had free access to feed and water until euthanasia, and no fasting was performed prior to slaughter to allow the collection of gastrointestinal contents for pH determination. Euthanasia was performed by electronarcosis followed by exsanguination, using five birds per treatment. Immediately after euthanasia, live body weight was recorded using a digital scale with four-decimal precision (0.0001 g) (Model Fruit Kitchen BFK40, Manufacturer: Bluetek, Paraná, Brazil).

For organ collection, the coelomic cavity was opened using dissection scissors, the organs were removed and emptied, and weighed on a digital scale with four-decimal precision (0.0001 g) (Model AUW220, Manufacturer: Shimadzu do Brasil, São Paulo, Brazil). These weights were used to calculate relative organ weight, expressed as a percentage of live body weight, using the formula: Relative organ weight (%) = (organ weight ÷ live body weight) × 100 (Watanabe, 2021).

For pH determination, 1 g of organ content was collected from euthanized birds, including cecum, duodenum, jejunum, ileum, gizzard, and proventriculus. The samples were mixed with 30 mL of distilled water in plastic universal containers, shaken, and allowed to rest for one minute. The pH was then measured using a portable digital pH meter (Model AKSO-AK90, Manufacturer: AKSO, Rio Grande do Sul, Brazil) (Coon et al., 1990).

### Histological and morphometric study

Following euthanasia, intestinal tissue samples (duodenum, jejunum, and ileum) were collected from five birds randomly selected from each treatment and immediately fixed in buffered formalin for 24 hours, followed by paraffin embedding. Sections of 5 μm thickness were prepared and stained with hematoxylin–eosin and Periodic Acid–Schiff (PAS). Digital images were captured using an Olympus BX-60 microscope coupled with a Zeiss AxioCam camera and Motic Image Plus 2.0 software (Model Olympus BX-60, Manufacturer: Evident).

Duodenal samples were collected 4 cm distal to the ventriculus; jejunal samples were taken from the middle region of the segment; ileal samples were taken from the terminal segment. All samples were sectioned transversely to allow visualization of intestinal villi and the lumen of the organ.

After histological processing, measurements were performed on 18 structures per tissue fragment for each bird, including villus height, villus width, and crypt depth. Villus height was measured from the base to the apex, villus width was measured at three different points along each villus, and crypt depth was measured from the base of its corresponding villus.

The villus-to-crypt ratio was calculated by dividing villus height by the corresponding crypt depth. Villus absorptive area was calculated by multiplying villus height by villus width.

### Quantification of goblet cells in the duodenum, jejunum, and ileum

Goblet cell quantification in the epithelium of the duodenum, jejunum, and ileum was performed using histological slides from five birds per treatment, stained with the Periodic Acid-Schiff (PAS) technique, which highlights goblet cells in magenta. Multiple digital images of the intestinal villi were captured at 40 × magnification for subsequent counting and analysis of the cells.

At least eight measurements per bird were randomly selected, and the intestinal epithelium was measured linearly over a length of 500 micrometers. Within these measured linear epithelial segments, the number of goblet cells was counted. From these results, the number of goblet cells per 500 micrometers of epithelium was determined for each treatment.

### Serum biochemistry

At the end of the fourth experimental cycle, prior to sample collection, birds were subjected to a 6-hour fasting period. Blood samples (approximately 4 mL) were collected from five birds per treatment by jugular vein puncture using sterile 13 × 0.4 mm needles. Blood was collected into dry tubes containing a clot activator (BD Vacutainer® Dry). Samples were allowed to rest at room temperature for 30 minutes to enable clot formation and were then centrifuged at 3,500 rpm for 10 minutes in a bench centrifuge (SL-702/RAF30, Solab, Piracicaba, SP, Brazil) to obtain the serum.

The separated serum was transferred to 2 mL Eppendorf-type microtubes and stored frozen until analysis. Biochemical analyses were performed at the Poultry Laboratory of the Federal University of Paraíba using commercial diagnostic kits (Labtest Diagnóstica S.A.®). The evaluated parameters included glucose (GLU; Ref. 133), triglycerides (TG; Ref. 87), cholesterol (CHOL; Ref. 1082), uric acid (UA; Ref. 140), urea (U; Ref. 1013), creatinine (CRE; Ref. 1010), aspartate aminotransferase (AST; Ref. 1009), alanine aminotransferase (ALT; Ref. 1008), gamma-glutamyl transferase (GGT; Ref. 1058), alkaline phosphatase (ALP; Ref. 1011), total protein (TP; Ref. 1085), albumin (ALB; Ref. 1007), calcium (Ca; Ref. 1084), and phosphorus (P; Ref. 12-200). All analyses were conducted using an automatic biochemical analyzer (Model SX-260, Manufacturer: Sinnowa, São Paulo, Brazil).

### Statistical analysis

Data were tested for homogeneity of variances and normality of residuals using SAS OnDemand. The experiment was conducted in a completely randomized design (CRD), and the data were subjected to analysis of variance (ANOVA) according to the following statistical model:

Yij=μ+Ti+εij

Where:

• Yij = observed response variable in the j-th replicate of treatment *i*
• μ = overall mean
• Ti = fixed effect of treatment *i*
• εij = random error associated with the observation, assumed to be normally distributed

When significant treatment effects were detected, means were compared using Tukey’s test (P < 0.05).

## Results

### Performance

Significant effects were observed for egg production (P = 0.014) and egg weight (P = 0.006) (Table 2). Birds in the 200AD2 treatment exhibited lower egg production compared with those in 500AD1 and 500AD2, with no differences among the remaining treatments. Egg weight was lower in birds subjected to 500AD2 than in 200AD1, while other treatments showed similar mean values.

**Table 2:** Productive performance of laying hens at 46 weeks of age supplemented with microencapsulated organic acids and phenolic compounds.

Treatments*	Variables⁎⁎						
	FI (g/day)	EP (%)	EW (g)	EM (g)	FCRM (g/g)	FCRD (g/dozen)	VIAB (%)
CD	118.73	91.48ab	62.44ab	57.08	2.12	1.57	97.00
200AD1	119.16	91.54ab	63.20a	57.09	2.11	1.59	98.00
500AD1	119.11	91.93a	62.52ab	57.44	2.10	1.58	98.00
200AD2	119.02	89.89b	62.45ab	56.49	2.11	1.57	98.00
500AD2	119.92	92.13a	62.09b	57.09	2.14	1.59	100.00
*P-value*	0.853	0.014	0.006	0.371	0.707	0.768	0.739
SEM	0.342	0.210	0.089	0.146	0.010	0.007	0.696

a,b Within a column, values with different letters differ statistically at 5%.

⁎ CD = Control diet; 200AD1 = 200 g/t additive 1; 500AD1 = 500 g/t additive 1; 200AD2 = 200 g/t additive 2; 500AD2 = 500 g/t additive 2.

⁎⁎ FI = Feed intake; EP = Egg production; EW = Egg weight; EM = Egg mass; FCRM = Feed conversion per egg mass; FCRD = Feed conversion per dozen eggs; VIAB = Viability. SEM = Standard error of the mean.

### Egg quality

Significant effects were observed for yolk color (P = 0.031), shell thickness (P = 0.001), yolk index (P = 0.001), specific gravity (P < 0.001), yolk pH (P = 0.009), and albumen pH (P < 0.001) (Table 3). Birds in the CD treatment exhibited lower mean yolk color compared with those in 200AD2, while no differences were observed relative to the other treatments. Shell thickness was highest in birds subjected to 200AD2 compared with CD, 200AD1, 500AD1, and 500AD2. Yolk index was lower in CD than in 200AD2 and 500AD2, with no significant differences compared with 200AD1 and 500AD1. Birds in the CD treatment also presented higher specific gravity than those in all other treatments. Regarding yolk pH, birds in 500AD1 and 200AD2 showed lower mean values than birds in 500AD2, without differing from CD and 200AD1. For albumen pH, birds in CD exhibited the highest mean value, whereas the lowest mean was observed in 200AD1, with intermediate values for the remaining treatments.

**Table 3:** Egg quality of laying hens at 46 weeks of age supplemented with microencapsulated organic acids and phenolic compounds.

Treatments*	Variables⁎⁎										
	YC	YCColor	ST (mm)	HU	YI	SG (g/cm^3^)	SP (%)	YP (%)	AP (%)	pH_Y	pH_A
CD	4.83	10.29b	0.403b	89.62	0.418b	1.081a	10.41	25.44	64.16	5.98ab	8.45a
200AD1	4.77	10.32ab	0.402b	89.78	0.421ab	1.076b	10.40	25.68	64.01	6.00ab	8.04d
500AD1	4.79	10.34ab	0.402b	89.87	0.425ab	1.072c	10.39	25.48	64.05	5.95b	8.21c
200AD2	4.67	10.50a	0.408a	89.54	0.426a	1.072c	10.35	25.56	64.07	5.92b	8.34b
500AD2	4.79	10.34ab	0.401b	90.06	0.428a	1.069c	10.34	25.65	63.94	6.07a	8.33b
*P-value*	0.354	0.031	0.001	0.598	0.001	<0.001	0.356	0.400	0.710	0.009	<0.001
SEM	0.025	0.022	0.001	0.111	0.001	0.004	0.013	0.046	0.049	0.013	0.011

a,b Within a column, values with different letters differ statistically at 5%.

⁎ CD = Control diet; 200AD1 = 200 g/t additive 1; 500AD1 = 500 g/t additive 1; 200AD2 = 200 g/t additive 2; 500AD2 = 500 g/t additive 2.

⁎⁎ YC = Yolk centering; YCColor = Yolk color; ST = Shell thickness; HU = Haugh unit; YI = Yolk index; SG = Specific gravity; SP = Shell percentage; YP = Yolk percentage; AP = Albumen percentage; pH_Y = Yolk pH; pH_A = Albumen pH. SEM = Standard error of the mean.

### Organ weights

Significant effects were observed for relative weights of gizzard (P = 0.033), small intestine (P = 0.040), pancreas (P = 0.043), proventriculus (P = 0.005), and spleen (P = 0.022) (Table 4). Birds subjected to 200AD2 exhibited higher mean relative gizzard weight than those in 500AD1 and 500AD2, without differing from CD and 200AD1. A similar pattern was observed for relative small intestine weight, in which birds in 200AD2 presented higher mean values compared with 200AD1, 500AD1, and 500AD2, with no difference relative to CD. Relative pancreas weight was higher in 200AD2 than in all other treatments. Birds in 200AD2 also exhibited higher relative proventriculus weight compared with CD and 200AD1, without differing from 500AD1 and 500AD2. Finally, relative spleen weight was greater in birds subjected to 200AD2 than in CD and 500AD1, with no significant differences relative to 200AD1 and 500AD2.

**Table 4:** Organ weights and abdominal fat of laying hens at 46 weeks of age supplemented with microencapsulated organic acids and phenolic compounds.

Treatments*	Variables^⁎⁎^							
	RGW (%)	RLW (%)	RSIW (%)	RLIW (%)	RFW (%)	RPW (%)	RPVW (%)	RSW (%)
CD	2.1421ab	2.1071	3.2831ab	1.0017	6.3407	0.2100b	0.4571c	0.1044b
200AD1	2.2744ab	2.0151	2.9704b	1.0243	5.3112	0.2046b	0.4817bc	0.1073ab
500AD1	1.9162b	1.9778	2.9787b	1.0093	4.7185	0.2021b	0.6030abc	0.1041b
200AD2	2.3604a	2.1135	3.4535a	0.9704	5.0231	0.2242a	0.7189a	0.1187a
500AD2	1.9266b	1.9951	2.9446b	0.9920	5.6364	0.2077b	0.6860ab	0.1074ab
*P-value*	0.033	0.181	0.040	0.796	0.179	0.043	0.005	0.022
SEM	0.049	0.022	0.055	0.014	0.211	0.002	0.023	0.001

a,b Within a column, values with different letters differ statistically at 5%. *CD = Control diet; 200AD1 = 200 g/t additive 1; 500AD1 = 500 g/t additive 1; 200AD2 = 200 g/t additive 2; 500AD2 = 500 g/t additive 2. **RGW = Relative gizzard weight; RLW = Relative liver weight; RSIW = Relative small intestine weight; RLIW = Relative large intestine weight; RFW = Relative fat weight; RPW = Relative pancreas weight; RPVW = Relative proventriculus weight; RSW = Relative spleen weight. SEM = Standard error of the mean.

### Organ pH

Among the measured organ pH values, only cecal pH showed a significant effect (P < 0.001) (Table 5). Birds subjected to 500AD1 exhibited lower mean cecal pH compared with those in CD, 200AD1, 200AD2, and 500AD2, with no differences among the latter treatments.

**Table 5:** Organ pH of laying hens at 46 weeks of age supplemented with microencapsulated organic acids and phenolic compounds.

Treatments*	Variables⁎⁎					
	pH_C	pH_D	pH_I	pH_J	pH_G	pH_P
CD	7.40a	6.28	7.24	6.30	5.08	5.20
200AD1	7.50a	6.46	7.52	6.46	5.14	5.08
500AD1	6.94b	6.30	7.24	6.28	4.26	4.46
200AD2	7.40a	6.28	6.98	6.60	4.60	4.96
500AD2	7.34a	6.16	7.44	6.44	4.46	4.70
*P-value*	<0.001	0.126	0.522	0.078	0.137	0.372
SEM	0.028	0.034	0.103	0.037	0.123	0.126

a,b Within a column, values with different letters differ statistically at 5%.

⁎ CD = Control diet; 200AD1 = 200 g/t additive 1; 500AD1 = 500 g/t additive 1; 200AD2 = 200 g/t additive 2; 500AD2 = 500 g/t additive 2.

⁎⁎ pH_C = Cecum pH; pH_D = Duodenum pH; pH_I = Ileum pH; pH_J = Jejunum pH; pH_G = Gizzard pH; pH_P = Proventriculus pH. SEM = Standard error of the mean.

### Duodenal histomorphometry

All evaluated variables were significantly affected: villus width (P = 0.001), villus height (P = 0.001), crypt depth (P < 0.001), villus:crypt ratio (P < 0.001), absorptive area (P = 0.022), and goblet cell counts (P < 0.001) (Table 6). Birds subjected to CD exhibited higher mean values of villus width compared with those in 200AD2 and 500AD2, without differing from the remaining treatments. Crypt depth was also highest in birds from the CD treatment, with means exceeding those in 200AD1, 500AD1, 200AD2, and 500AD2. Villus height was highest in 500AD1, differing from most treatments except 500AD2, which presented similar values. The villus:crypt ratio was highest in 500AD2, exceeding the values in CD, 200AD1, and 200AD2, without differing from 500AD1. Absorptive area was highest in 500AD1 compared with 200AD2, without differing from the remaining treatments. Goblet cell counts were lowest in birds subjected to 200AD1, with higher counts observed in CD, 500AD1, 200AD2, and 500AD2.

**Table 6:** Duodenal histomorphometry of laying hens at 46 weeks of age supplemented with microencapsulated organic acids and phenolic compounds.

Treatments*	Variables⁎⁎					
	VW	VH	CD	VCR	AA	GC
	(μm)	(μm)	(μm)	(μm)	(μm²)	
CD	273.94a	1383.57c	119.29a	11.67d	3792ab	49.77a
200AD1	265.50ab	1455.62bc	88.16b	16.53bc	3860ab	39.65b
500AD1	249.74abc	1655.65a	86.27b	19.18ab	4139a	48.95a
200AD2	245.24bc	1366.87c	83.85b	16.36c	3352b	50.52a
500AD2	225.11c	1618.65ab	79.17b	20.55a	3644ab	47.92a
*P-value*	0.004	0.001	<0.001	<0.001	0.022	<0.001
SEM	2.958	18.916	1.352	0.296	6796.0	0.428

a,b Within a column, values with different letters differ statistically at 5%.

⁎ CD = Control diet; 200AD1 = 200 g/t additive 1; 500AD1 = 500 g/t additive 1; 200AD2 = 200 g/t additive 2; 500AD2 = 500 g/t additive 2.

⁎⁎ VW = Villus width; VH = Villus height; CD = Crypt depth; VCR = Villus:crypt ratio; AA = Absorptive area; GC = Goblet cells. SEM = Standard error of the mean.

### Jejunal histomorphometry

All evaluated variables were significantly affected: villus width (P = 0.003), villus height (P < 0.001), crypt depth (P < 0.001), villus:crypt ratio (P < 0.001), absorptive area (P = 0.0202), and goblet cell counts (P < 0.001) (Table 7). Birds subjected to 500AD1 exhibited higher mean values of villus width compared with those in CD and 500AD2, without differing from the remaining treatments. Villus height was higher in birds from the CD and 500AD2 treatments compared with the other treatments. Crypt depth was highest in 200AD1, exceeding the values in 500AD1, 200AD2, and 500AD2, without differing from CD. Villus:crypt ratio was greater in birds from CD, 200AD2, and 500AD2 compared with those in 200AD1 and 500AD1. Absorptive area was highest in 500AD2, with means exceeding those in 500AD1, without differing from CD, 200AD1, and 200AD2. Goblet cell counts were lowest in CD, with higher counts observed in 200AD1, 500AD1, 200AD2, and 500AD2.

**Table 7:** Jejunal histomorphometry of laying hens at 46 weeks of age supplemented with microencapsulated organic acids and phenolic compounds.

Treatments*	Variables⁎⁎					
	VW	VH	CD	VCR	AA	GC
	(μm)	(μm)	(μm)	(μm)	(μm²)	
CD	157.00c	1169.88a	80.90ab	14.51a	1839ab	47.52b
200AD1	177.71ab	955.83b	81.82a	11.69b	1697ab	55.05a
500AD1	179.89a	894.59b	75.52bc	11.85b	1602b	53.35a
200AD2	178.32ab	1012.44b	69.96c	14.48a	1805ab	54.60a
500AD2	157.97bc	1213.95a	73.55c	16.51a	1914a	51.80a
*P-value*	0.003	<0.001	<0.001	<0.001	0.020	<0.001
SEM	2.161	14.095	0.611	0.223	2846.32	0.387

a,b Within a column, values with different letters differ statistically at 5%.

⁎ CD = Control diet; 200AD1 = 200 g/t additive 1; 500AD1 = 500 g/t additive 1; 200AD2 = 200 g/t additive 2; 500AD2 = 500 g/t additive 2.

⁎⁎ VW = Villus width; VH = Villus height; CD = Crypt depth; VCR = Villus:crypt ratio; AA = Absorptive area; GC = Goblet cells. SEM = Standard error of the mean.

### Ileal histomorphometry

All analyzed variables were significantly affected: villus width (P < 0.001), villus height (P < 0.001), crypt depth (P < 0.001), and villus:crypt ratio (P < 0.001) (Table 8). Birds subjected to 200AD2 exhibited higher mean values of villus width compared with those in CD, 200AD1, and 500AD2, and were similar to those in 500AD1. Villus height was highest in birds from the 500AD2 treatment, exceeding the means of CD, 200AD1, 500AD1, and 200AD2. Crypt depth was higher in birds from 200AD1 compared with CD, 500AD1, 200AD2, and 500AD2. Villus:crypt ratio was highest in 500AD2, with mean values greater than those observed in CD, 200AD1, 500AD1, and 200AD2.

**Table 8:** Ileal histomorphometry of laying hens at 46 weeks of age supplemented with microencapsulated organic acids and phenolic compounds.

Treatments*	Variables⁎⁎					
	VW	VH	CD	VCR	AA	GC
	(μm)	(μm)	(μm)	(μm)	(μm²)	
CD	153.53b	455.29bc	66.34b	6.87bc	6991	56.62
200AD1	133.43c	505.51b	73.39a	6.90bc	6767	55.67
500AD1	172.54ab	395.47c	63.46b	6.27c	6832	56.90
200AD2	178.45a	446.48bc	55.92c	7.99b	7963	55.05
500AD2	112.74d	608.41a	46.69d	13.04a	6879	54.89
*P-value*	<0.001	<0.001	<0.001	<0.001	0.199	0.246
SEM	2.083	6.836	0.674	0.135	1605.24	0.346

a,b Within a column, values with different letters differ statistically at 5%.

⁎ CD = Control diet; 200AD1 = 200 g/t additive 1; 500AD1 = 500 g/t additive 1; 200AD2 = 200 g/t additive 2; 500AD2 = 500 g/t additive 2.

⁎⁎ VW = Villus width; VH = Villus height; CD = Crypt depth; VCR = Villus:crypt ratio; AA = Absorptive area; GC = Goblet cells. SEM = Standard error of the mean.

**Fig. 2:**
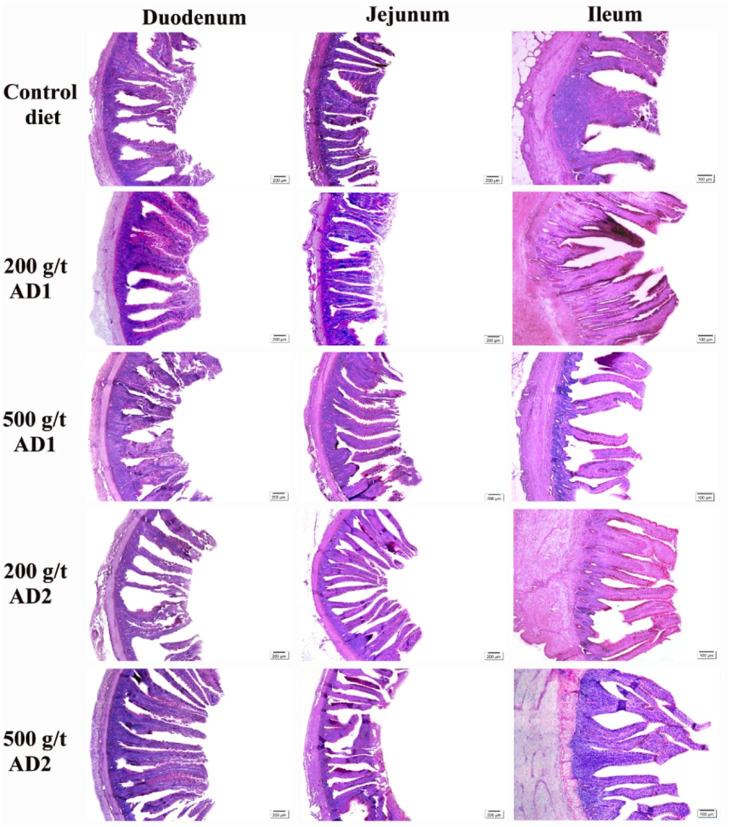
Photomicrographs of transverse sections of the duodenum, jejunum, and ileum from the small intestine of 46-week-old Novogen Brown laying hens fed diets supplemented with different levels of microencapsulated organic acids and phenolic compounds. Staining: (hematoxilina-eosina e PAS). Staining: PAS. Scale bars: duodenum, jejunum: 200 µm and ileum: 100 µm. CD = Control diet; 200AD1 = 200 g/t additive 1 (Palm oil, citric acid (min.) 65 g/kg, fumaric acid (min.) 130 g/kg, phosphoric acid (min.) 65 g/kg, malic acid (min.) 65 g/kg, lauric acid (min.) 120 mg/kg, thymol (min.) 5 g/kg, carvacrol (min.) 5 g/kg, cineole (min.) 5 g/kg); 500AD1 = 500 g/t additive 1; 200AD2 = 200 g/t additive 2 (Palm oil, citric acid (min.) 65 g/kg, fumaric acid (min.) 130 g/kg, phosphoric acid (min.) 65 g/kg, malic acid (min.) 65 g/kg, lauric acid (min.) 120 mg/kg, thymol (min.) 20 g/kg, carvacrol (min.) 10 g/kg); 500AD2 = 500 g/t additive 2.

Fig. 2. illustrates the overall histomorphological patterns in the duodenum, jejunum, and ileum of laying hens fed diets supplemented with different levels of microencapsulated organic acids and phenolic compounds. The figure highlights treatment-related variations in villus height and width, crypt depth, villus-to-crypt ratio, and goblet cell numbers, serving as a visual complement to the data reported in Table 6, Table 7, Table 8.

### Serum biochemical parameters

Significant effects were observed for uric acid (P = 0.001), GGT (P = 0.002), calcium (P = 0.028), and phosphorus (P = 0.005) (Table 9). Birds subjected to the 200AD2 treatment exhibited higher mean values of uric acid compared with those in CD, 200AD1, and 500AD2, and showed similar values to birds in 500AD1. Regarding GGT, birds in 500AD2 had higher mean values than those in CD and 200AD1, without differing from the remaining treatments. For calcium, birds in 200AD1 and 200AD2 presented higher mean values compared with CD, while no differences were observed relative to 500AD1 and 500AD2. Phosphorus mean values were higher in birds from 200AD1 and 500AD1 compared with CD, with no statistical differences relative to 200AD2 and 500AD2.

**Table 9:** Serum biochemistry of laying hens at 46 weeks of age supplemented with microencapsulated organic acids and phenolic compounds.

Variables^⁎⁎^	Treataments*					*P-value*	SEM
	CD	200AD1	500AD1	200AD2	500AD2		
GLU, mg/dl	137.00	154.20	137.80	132.40	148.00	0.580	4.666
TG, mg/dl	1784.3	2448.0	2323.0	1849.0	1836.0	0.252	111.01
CHOL, mg/dl	139.60	106.80	143.40	105.00	117.00	0.110	5.459
UA, mg/dl	4.40b	4.40b	5.00ab	5.75a	4.00b	0.001	0.113
U, mg/dl	7.20	6.00	7.60	7.60	7.40	0.190	0.229
CRE, mg/dl	0.312	0.344	0.292	0.350	0.384	0.077	0.010
AST, U/L	162.00	154.00	158.20	169.60	183.80	0.319	4.674
ALT, U/L	5.75	5.20	6.20	5.20	5.00	0.395	0.213
GGT, U/L	22.50bc	16.00c	28.25ab	32.25ab	33.25a	0.002	1.035
ALP, U/L	352.00	249.75	249.80	291.00	305.40	0.659	24.505
TP, g/dl	4.80	4.40	4.80	4.40	4.40	0.298	0.068
ALB, g/dl	2.40	2.40	2.00	2.20	2.00	0.052	0.027
Ca, mg/dl	17.35b	22.76a	18.94ab	23.41a	18.70ab	0.028	0.644
P, mg/dl	3.905b	5.480a	5.408a	4.885ab	4.212ab	0.005	0.138

a,b Within a row, values with different letters differ statistically at 5%. *CD = Control diet; 200AD1 = 200 g/t additive 1; 500AD1 = 500 g/t additive 1; 200AD2 = 200 g/t additive 2; 500AD2 = 500 g/t additive 2. **GLU = Glucose; TG = Triglycerides; CHOL = Cholesterol; UA = Uric acid; U = Urea; CRE = Creatinine; AST = Aspartate aminotransferase; ALT = Alanine aminotransferase; GGT = Gamma-glutamyltransferase; ALP = Alkaline phosphatase; TP = Total protein; ALB = Albumin; Ca = Calcium; P = Phosphorus. SEM = Standard error of the mean.

## Discussion

### Performance

The supplementation of organic acids and essential oils in poultry diets has been widely investigated as an alternative to antibiotic growth promoters, mainly due to their effects on intestinal microbiota, nutrient digestibility, and intestinal mucosal integrity (Khan and Iqbal, 2016; Stamilla et al., 2020). These compounds exhibit antimicrobial and antioxidant activities capable of modulating the intestinal environment, reducing colonization by pathogenic microorganisms, and promoting beneficial bacteria, which may improve digestive efficiency and nutrient utilization from the diet (Kunz et al., 2011; Islam et al., 2022).

In the present study, different levels of microencapsulated additives influenced the productive performance of laying hens, particularly egg production and egg weight. Similar results were reported by Konca et al. (2024), who observed increased egg production and egg mass, as well as greater eggshell thickness in birds supplemented with organic acids and essential oils compared with the control group.

The lower production observed at the lowest inclusion level of AD2 suggests that the concentration or composition of the additive may not have been sufficient to promote effective modulation of the gastrointestinal environment, which may have reduced the availability of metabolizable energy and amino acids required for egg synthesis. Regarding egg weight, although the 500AD2 treatment presented a lower average (62.09 g), the Novogen Brown management guide indicates an expected average egg weight of 60.90 g for birds at 46 wk of age (Novogen, 2022), demonstrating that all evaluated treatments remained within or above the breed standard.

On the other hand, the higher inclusion levels (500AD1 and 500AD2) indicate greater metabolic efficiency and improved nutrient utilization, possibly associated with the gradual release of bioactive compounds throughout the digestive tract. Microencapsulation may contribute to this effect by protecting the active compounds from early degradation and allowing their progressive release in more distal regions of the intestine, increasing their bioavailability and enhancing their physiological effects (Abdelli et al., 2021; Moharreri et al., 2021).

These results may be related to the combined action of organic acids and essential oils in modulating the intestinal microbiota, reducing luminal pH, and stimulating the activity of endogenous digestive enzymes, factors that favor the digestion and absorption of nutrients essential for egg formation, such as amino acids, lipids, and minerals (Menten et al., 2014; Khan and Iqbal, 2016). Similarly, Huang et al. (2024) reported that supplementation with encapsulated essential oils and organic acids improves performance and the intestinal microenvironment, associating these effects with positive changes in intestinal morphology, increased digestive enzyme activity, higher populations of lactic acid bacteria, and greater butyric acid concentration in the cecum, which also contributes to intestinal barrier integrity and maintenance of immune homeostasis.

According to Lee et al. (2025), the inclusion of organic acids in the diet may improve productive performance and feed efficiency in poultry. These effects are associated with improved intestinal health, increased nutrient digestibility and absorption, as well as microbiota stabilization and reduction of intestinal pH. Similar findings were reported by Wang et al. (2022), who observed an increase in laying rate in hens supplemented with microencapsulated essential oils, accompanied by improvements in intestinal morphology and efficiency of nutrient digestion and absorption. However, productive responses are not always consistent. Stamilla et al. (2020) did not observe significant effects on some productive parameters of laying hens supplemented with phytogenic additives and organic acids, indicating that factors such as inclusion level, additive composition, bird age, and management conditions may influence the magnitude of the responses.

### Egg quality

In the present study, the changes observed in egg quality indicate possible effects of microencapsulated additives on nutrient absorption and bird metabolism. The greater yolk color intensity observed in supplemented birds suggests increased deposition of liposoluble pigments, such as carotenoids, which may be associated with a more balanced intestinal environment and improved lipid digestibility, favoring the transport of these compounds to the yolk (Yasin et al., 2025).

Considering the composition of the additives used, compounds such as carvacrol and thymol may contribute to improvements in egg quality and intestinal health. In addition, medium-chain fatty acids, such as lauric acid, may enhance lipid digestion and absorption, facilitating the deposition of liposoluble pigments in the yolk (Wang et al., 2022; Liu et al., 2026). In contrast, the lower yolk coloration observed in the control group may indicate lower efficiency in the absorption of these compounds, without necessarily affecting egg formation, but limiting the natural enrichment of the yolk.

Eggshell thickness and yolk index are relevant indicators of egg quality and may reflect the effects of supplementation on mineralization and structural composition. The greater eggshell thickness observed in some treatments suggests improved utilization of essential minerals for shell formation, particularly calcium and phosphorus. Organic acids present in the additives, such as citric, fumaric, malic, and phosphoric acids, may reduce gastrointestinal pH, increasing the solubility and availability of these minerals and favoring their intestinal absorption and deposition in the eggshell (Sakomura et al., 2014; Vargas-Rodriguez et al., 2015). Furthermore, fumaric acid acts as an intermediate in cellular energy metabolism and may indirectly contribute to processes involved in egg formation.

Changes in the yolk index may also reflect alterations in the metabolic efficiency of birds, particularly in the deposition of lipids and proteins. Adequate values of this index are associated with the integrity of the vitelline membrane and the maintenance of internal egg quality (Alves et al., 2017). Therefore, improvements in this parameter may indicate greater efficiency in directing nutrients toward egg formation.

Regarding specific gravity, the higher values observed in the control group may represent a compensatory physiological response of birds in the absence of supplementation. Specific gravity is directly related to eggshell quality and strength and is considered adequate when higher than approximately 1.085 g/cm³ (Peebles and McDaniel, 2013). Thus, even in the absence of additives, physiological mechanisms may act to maintain the structural integrity of the egg, although without the additional potential effects of bioactive compounds.

Variations in yolk and albumen pH also indicate possible effects of supplementation on metabolism and acid–base balance. The pH of freshly laid albumen generally ranges from 7.6 to 7.9, whereas yolk pH is typically close to 6.0 (Feddern et al., 2017). Changes in these parameters may be related to CO₂ loss through the pores of the eggshell and to biochemical transformations that occur after oviposition, affecting internal egg quality (Solomon, 1997). In this context, the inclusion of organic acids in the diet may influence metabolism and intestinal acid–base balance, reflecting changes in the physicochemical characteristics of the egg.

These findings are consistent with studies demonstrating that the inclusion of organic acids in laying hen diets can improve eggshell quality, mainly due to increased mineral availability and absorption (Soltan, 2008; Gül et al., 2014). In addition, phenolic compounds present in essential oils, such as thymol and carvacrol, exhibit antioxidant and antimicrobial properties that may contribute to the lipid stability of the yolk and to the maintenance of internal egg quality (Gholami-Ahangaran et al., 2022).

Overall, the results indicate that the composition and inclusion level of microencapsulated additives may influence egg quality parameters, such as yolk color, eggshell thickness, yolk index, specific gravity, and pH. The combination of different organic acids, lauric acid, and phenolic compounds may act synergistically in improving nutrient digestibility, mineral absorption, and antioxidant protection, contributing to improvements in egg quality without compromising productive performance. Similar findings were reported by Moharreri et al. (2021), who observed improvements in egg quality traits in birds supplemented with plant-derived bioactive compounds.

### Organ weights

In the present study, supplementation influenced the relative weight of some digestive and immune organs, such as the small intestine, pancreas, proventriculus, and spleen. These changes may reflect morphophysiological adaptations of the gastrointestinal tract in response to the presence of bioactive compounds in the diet, which are capable of modulating digestive and metabolic processes in birds. The development of these structures is directly related to digestive efficiency and nutrient utilization, since the digestive tract is responsible for the digestion and absorption of dietary components (Sakomura et al., 2014; Reece, 2017).

The greater relative weight of the small intestine may indicate increased absorptive capacity, as this segment contains villi responsible for expanding the surface area available for nutrient absorption (Rowe and Reece, 2020). Similarly, the increased relative weight of the pancreas and proventriculus may be associated with enhanced chemical digestion, involving greater secretion of digestive enzymes and hydrochloric acid (Sakomura et al., 2014; Araújo and Zanetti, 2019).

In addition, the higher relative weight of the spleen may be associated with modulation of the immune response, considering that this organ plays an important role in host defense and may respond to changes in intestinal microbiota and in the physiological environment of birds (Du et al., 2020; Khan et al., 2022).

Overall, changes in the relative weight of digestive organs may represent physiological adjustments associated with digestive activity and microbial balance within the gastrointestinal tract. The development of these structures may be influenced by diet composition and by the presence of bioactive compounds that stimulate enzymatic secretion, digestion, and metabolism in birds (Sakomura et al., 2014; Macari and Maiorka, 2017; Islam et al., 2022). Phenolic compounds present in essential oils, such as thymol and carvacrol, may also contribute to the modulation of metabolic processes and improvement of nutrient digestibility, promoting greater physiological efficiency and improved nutrient utilization (Du et al., 2020; Abdelli et al., 2021).

Similar results were reported by Wang et al. (2019), who observed that structural changes in the digestive tract associated with supplementation of bioactive compounds may increase digestive efficiency and nutrient utilization. Likewise, Dibakoane et al. (2025) highlighted that improvements in intestinal health may promote more efficient immune responses without inducing excessive inflammatory processes. Thus, the observed results suggest that supplementation with 200AD2 may promote structural and functional adjustments in digestive and immune organs, contributing to improved digestion, nutrient absorption, and maintenance of bird health.

### Organ pH

The pH of different segments of the digestive tract is an important indicator of physiological conditions and intestinal microbial balance. In the present study, only the cecal pH differed significantly among treatments. Birds receiving the 500AD1 treatment exhibited a lower cecal pH compared with the other treatments, indicating greater acidification in this segment.

The cecum is one of the main sites of microbial fermentation in the avian gastrointestinal tract, where the degradation of undigested substrates originating from the anterior portions of the intestine occurs. This process results in the production of metabolites such as short-chain fatty acids (SCFA), which play an important role in intestinal physiology and in maintaining host energy homeostasis (Sakomura et al., 2014; Macari and Maiorka, 2017; Du et al., 2020). Thus, the reduction in cecal pH observed in the 500AD1 treatment may be associated with increased fermentative activity of the microbiota and, consequently, greater production of these metabolites.

The decrease in cecal pH may also be related to the direct action of organic acids present in the microencapsulated additive. These compounds reduce gastrointestinal pH and modulate intestinal microbiota, favoring the growth of beneficial bacteria while inhibiting the colonization of pathogenic microorganisms (Dibner and Buttin, 2002; Menten et al., 2014). As a result, a more stable and functional intestinal environment is established.

Similar findings were reported by Khan and Iqbal (2016), who observed an association between reduced intestinal pH and increased production of short-chain fatty acids in birds supplemented with acidifying additives. Broom (2015) also reported that organic acids can optimize the intestinal environment and regulate the expression of bacterial virulence genes. Although pH directly influences microbial growth, the antimicrobial effect of these compounds is mainly related to their undissociated form, which is considered the most active fraction in inhibiting bacterial development.

The absence of significant differences in the pH of the other digestive segments suggests that the effects of supplementation were more evident in the distal intestine. This result may be related to the use of microencapsulation, which allows the gradual release of bioactive compounds along the gastrointestinal tract. This technology protects volatile substances, such as organic acids and essential oils, during feed processing and prevents their dissociation or early absorption in the initial portions of the digestive tract, thereby favoring their action in the distal intestinal regions (Michiels et al., 2008; Jadhao et al., 2019). In addition, the progressive release of these compounds may enhance their effects on intestinal morphology and nutrient utilization (Abdelli et al., 2021).

Overall, the results indicate that microencapsulated supplementation, particularly in the 500AD1 treatment, was able to modify the cecal environment through pH reduction, which may favor the fermentative activity of beneficial microbiota, increase the production of short-chain fatty acids, and contribute to the maintenance of intestinal health and digestive efficiency in birds.

### Histology

The intestinal histomorphometry results indicated that supplementation with the additives promoted changes in intestinal mucosal morphology compared with the control treatment, particularly in villus height, crypt depth, and the villus:crypt ratio. Villus height is directly associated with the absorptive capacity of the small intestine, as more developed structures increase the surface area available for nutrient absorption. In contrast, deeper crypts indicate greater proliferative activity of the intestinal epithelium and increased energy demand for cellular renewal (Walton et al., 2016; Reece, 2017).

In the duodenum, the differences observed among treatments suggest that supplementation may promote villus development and contribute to a better balance between nutrient absorption and mucosal cell renewal. This segment plays an important role in the initial stages of digestion, as it receives pancreatic and biliary secretions responsible for nutrient breakdown (Reece, 2017).

In the jejunum, considered the main site of digestion and absorption in poultry, absorptive efficiency is directly related to the surface area of the intestinal mucosa, which is primarily determined by villus height and width. Therefore, positive changes in these parameters may favor greater utilization of ingested nutrients (Walton et al., 2016). Miao et al. (2022) reported that supplementation with organic acids improves the ratio between villus height and crypt depth, indicating greater absorptive efficiency and improved nutrient allocation for egg production and egg quality. Similarly, Johnson et al. (2023) observed that microencapsulated mixtures of organic acids and plant-derived compounds may promote a more anti-inflammatory intestinal phenotype and reduce inflammatory signaling in the jejunum, in addition to improving immunometabolic responses in the ileum.

Regarding goblet cells, a greater presence was observed in the more distal segments of the small intestine. These cells are responsible for mucin production, the main component of intestinal mucus, which acts as a physical barrier protecting the epithelium from mechanical damage caused by digesta and from the adhesion of pathogenic microorganisms (Abrahamsohn, 2016).

The changes observed in intestinal morphology may be associated with the antimicrobial and microbiota-modulating effects promoted by organic acids and phenolic compounds present in the additives. These compounds may reduce pathogenic microbial load, stimulate digestive secretions, and enhance nutrient absorption, thereby contributing to the maintenance of intestinal mucosal integrity and microbial balance (Macari and Maiorka, 2017; Abdelli et al., 2021).

Furthermore, studies have shown that supplementation with organic acids and essential oils can promote structural improvements in the intestine, including increased villus height and greater absorptive surface area of the mucosa. Wang et al. (2019) and Stamilla et al. (2020) reported that the inclusion of these compounds in diets for laying hens resulted in favorable morphological changes, such as more developed villi and improved organization of the intestinal mucosa.

Supplementation with microencapsulated organic acids and phenolic compounds may contribute to the development and maintenance of intestinal mucosal structure, promoting epithelial integrity and absorptive efficiency of the small intestine. These effects may positively influence intestinal health and productive performance in birds (Abdelli et al., 2021; Islam et al., 2022).

### Serum biochemistry

Serum biochemical parameters are widely used to assess the physiological and metabolic status of birds, providing information on protein metabolism, liver function, and mineral balance. In the present study, differences among treatments were observed for uric acid, gamma-glutamyl transferase (GGT), calcium, and phosphorus, indicating that supplementation with microencapsulated organic acids and phenolic compounds may influence important metabolic processes in laying hens.

Uric acid is the main end product of nitrogen metabolism in birds and is directly related to protein utilization and amino acid catabolism. Changes in its serum concentration may reflect alterations in protein digestibility, amino acid utilization efficiency, or renal excretion (Donsbough et al., 2010). The differences observed among treatments may be associated with the effects of organic acids on nutrient digestibility and modulation of intestinal microbiota. These compounds reduce gastrointestinal pH and inhibit pathogenic microorganisms, promoting a more stable intestinal environment and improving the utilization of dietary proteins (Sakomura et al., 2014).

Gamma-glutamyl transferase (GGT) is an enzyme associated with hepatic metabolism and the transport of amino acids across cellular membranes and is commonly used as an indicator of liver function in birds. Variations in its serum activity may reflect metabolic changes in the liver or physiological responses to nutritional interventions (Silvestre-Ferreira, 2025). The differences observed suggest a possible influence of the additives on hepatic metabolism. Phenolic compounds present in essential oils exhibit antioxidant and anti-inflammatory properties that may contribute to liver tissue protection and modulation of metabolic activity. In addition, organic acids act as acidifying agents in the digestive tract, reducing pH in the initial segments and enhancing pepsin activity. This effect may intensify protein digestion and exert bactericidal or bacteriostatic effects, hindering the adhesion of pathogenic bacteria to the intestinal mucosa and increasing nutrient availability. Because they are weak acids and partially dissociated, they may also act as antibacterial agents, immune modulators, and growth promoters (Abdelli et al., 2021; Khan et al., 2022).

Serum concentrations of calcium and phosphorus are particularly relevant in laying hens due to the role of these minerals in eggshell formation and maintenance of bone metabolism. Calcium is the main structural component of the eggshell and participates in physiological processes such as muscle contraction, nerve transmission, and cellular signaling. Phosphorus, in turn, plays an important role in bone mineralization and in metabolic processes related to energy production and cellular synthesis. Changes in intestinal morphology, such as an increase in mucosal absorptive surface area, may favor digestion and absorption of nutrients, including minerals (Sakomura et al., 2014).

In this context, citric acid present in the composition of the additives evaluated in this study may contribute to greater mineral digestibility, either through effects on intestinal morphology or through the formation of chelated complexes that increase the availability and retention of minerals such as calcium, iron, copper, and magnesium (Sakomura et al., 2014). In addition, Vargas-Rodriguez et al. (2015) reported that citric acid may reduce excretion and increase the digestibility of phosphorus, nitrogen, and calcium, in addition to enhancing the response to phytase by influencing the excretion of these nutrients.

Supplementation with microencapsulated organic acids and phenolic compounds may influence protein metabolism, hepatic activity, and mineral balance in laying hens, possibly through modulation of intestinal microbiota, improvement in nutrient digestibility, and greater efficiency in mineral absorption. Similar results were reported by Orzuna-Orzuna and Lara-Bueno (2023), who observed increased serum levels of superoxide dismutase and glutathione peroxidase in birds supplemented with essential oils, indicating greater antioxidant capacity and improved control of free radicals in the organism.

**Fig. 3:**
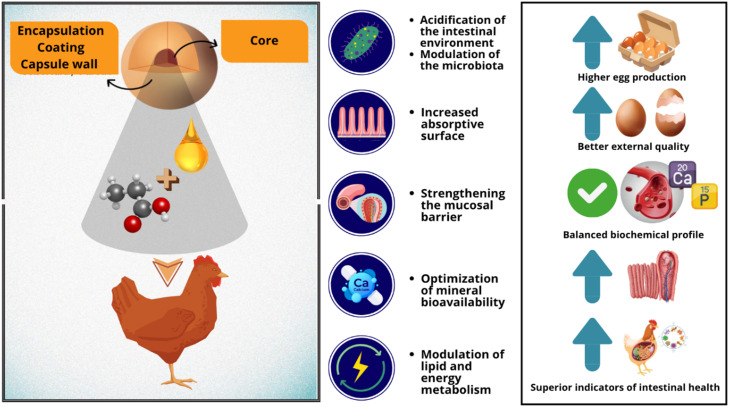
Mechanisms of action of microencapsulated acidifiers and phenolic compounds in laying hens. Illustrative scheme showing the effects of microencapsulation on intestinal integrity, nutrient absorption, and metabolism. The combined action of these compounds promotes microbiota balance and improved mineral and energy utilization, resulting in enhanced egg production and quality.

Taken together, the results of the present study indicate that supplementation with microencapsulated organic acids and phenolic compounds may influence multiple physiological processes in laying hens, including intestinal microbiota balance, digestive efficiency, intestinal morphology, and metabolic responses. The gradual release of bioactive compounds along the gastrointestinal tract may promote a more stable intestinal environment, enhancing nutrient digestion and absorption and contributing to improvements in productive performance and egg quality. A conceptual overview of the possible mechanisms involved in these responses is presented in Fig. 3.

## Conclusion

In summary, supplementation with microencapsulated organic acids and phenolic compounds modulated productive performance, egg quality, and intestinal physiology in laying hens, indicating that these bioactive compounds can influence digestive functionality and metabolic responses. Higher inclusion levels, particularly 500AD2, were associated with more favorable intestinal morphometric responses and productive outcomes, whereas 200AD2 appeared to promote structural adaptations of the gastrointestinal tract. These findings highlight the potential of microencapsulated acidifier–phytogenic blends as nutritional strategies to support intestinal functionality and productive efficiency in laying hens. Further studies should clarify the mechanisms involved and evaluate the consistency of these responses under different production conditions.

## CRediT authorship contribution statement

**Paloma Eduarda Lopes de Souza:** Writing – review & editing, Writing – original draft, Project administration, Methodology, Investigation, Formal analysis, Data curation, Conceptualization. **Adiel Vieira de Lima:** Writing – review & editing, Formal analysis, Data curation, Conceptualization. **Carlos Henrique do Nascimento:** Investigation, Formal analysis, Data curation, Conceptualization. **Aline Beatriz Rodrigues:** Data curation, Conceptualization. **Amanda Fabrício Dantas de Lima:** Data curation, Conceptualization. **Cleber Franklin Santos de Oliveira:** Data curation, Conceptualization. **Edijanio Galdino da Silva:** Data curation, Conceptualization. **Apolônio Gomes Ribeiro:** Writing – review & editing, Data curation, Conceptualization. **Ricardo Romão Guerra:** Writing – review & editing, Formal analysis, Data curation, Conceptualization. **Danilo Teixeira Cavalcante:** Formal analysis, Data curation, Conceptualization. **Isabelle Naemi Kaneko:** Formal analysis, Data curation, Conceptualization. **Andreia Massuquetto:** Resources, Conceptualization. **Thiago Pereira Ribeiro:** Resources, Conceptualization. **Lucas Rannier Ribeiro Antonino Carvalho:** Writing – review & editing, Resources, Formal analysis, Data curation, Conceptualization. **Fernando Guilherme Perazzo Costa:** Writing – review & editing, Writing – original draft, Validation, Supervision, Resources, Project administration, Methodology, Investigation, Funding acquisition, Formal analysis, Data curation, Conceptualization.

## Disclosures

The authors declare that they have no other conflicts of interest.
